# Differential effects of diet- and genetically-induced brain insulin resistance on amyloid pathology in a mouse model of Alzheimer’s disease

**DOI:** 10.1186/s13024-019-0315-7

**Published:** 2019-04-12

**Authors:** Tomoko Wakabayashi, Kazuki Yamaguchi, Kentaro Matsui, Toshiharu Sano, Tetsuya Kubota, Tadafumi Hashimoto, Ayako Mano, Kaoru Yamada, Yuko Matsuo, Naoto Kubota, Takashi Kadowaki, Takeshi Iwatsubo

**Affiliations:** 1 Department of Neuropathology, Graduate School of Medicine, The University of Tokyo, 7-3-1 Hongo, Bunkyo-ku, Tokyo, 113-0033 Japan; 2 Department of Innovative Dementia Prevention, Graduate School of Medicine, The University of Tokyo, Tokyo, 113-0033 Japan; 3 Laboratory for Intestinal Ecosystem, RIKEN Center for Integrative Medical Sciences, Kanagawa, 230-0045 Japan; 4 Department of Diabetes and Metabolic Diseases, Graduate School of Medicine, The University of Tokyo, Tokyo, 113-8655 Japan; 5 Department of Clinical Nutrition Therapy, The University of Tokyo, Tokyo, 113-8655 Japan; 6 Department of Prevention of Diabetes and Lifestyle-Related Diseases, Graduate School of Medicine, The University of Tokyo, Tokyo, 113-8655 Japan; 7 Department of Metabolism and Nutrition, Mizonokuchi Hospital, Faculty of Medicine, Teikyo University, Tokyo, 213-8507 Japan

**Keywords:** Alzheimer’s disease, Aβ, Type 2 diabetes mellitus, Insulin resistance, Dietary intervention

## Abstract

**Background:**

Based on epidemiological and experimental studies, type 2 diabetes mellitus (T2DM), especially insulin resistance that comprises the core mechanism of T2DM, has been recognized as a significant risk factor for Alzheimer’s disease (AD). Studies in humans and diabetic AD model mice have indicated a correlation between insulin resistance and increased amyloid deposition in the brain. Paradoxically, mice with targeted disruption of genes involved in the insulin signaling pathway showed protective effects against the AD-related pathology. These conflicting observations raise an issue as to the relationship between dysregulation of insulin signaling and AD pathophysiology.

**Methods:**

To study the causal relations and molecular mechanisms underlying insulin resistance-induced exacerbation of amyloid pathology, we investigated the chronological changes in the development of insulin resistance and amyloid pathology in two independent insulin-resistant AD mouse models, i.e., long-term high-fat diet (HFD) feeding and genetic disruption of *Irs2*, in combination with dietary interventions. In addition to biochemical and histopathological analyses, we examined the in vivo dynamics of brain amyloid-β (Aβ) and insulin by microdialysis technique.

**Results:**

HFD-fed diabetic AD model mice displayed a reduced brain response to peripheral insulin stimulation and a decreased brain to plasma ratio of insulin during the hyperinsulinemic clamp. Diet-induced defective insulin action in the brain was accompanied by a decreased clearance of the extracellular Aβ in vivo and an exacerbation of brain amyloid pathology. These noxious effects of the HFD both on insulin sensitivity and on Aβ deposition in brains were reversibly attenuated by dietary interventions. Importantly, HFD feeding accelerated Aβ deposition also in the brains of IRS-2-deficient AD mice.

**Conclusions:**

Our results suggested a causal and reversible association of brain Aβ metabolism and amyloid pathology by diet-dependent, but not genetically-induced, insulin-resistance. These observations raise the possibility that the causal factors of insulin resistance, e.g., metabolic stress or inflammation induced by HFD feeding, but not impaired insulin signaling per se, might be directly involved in the acceleration of amyloid pathology in the brain.

**Electronic supplementary material:**

The online version of this article (10.1186/s13024-019-0315-7) contains supplementary material, which is available to authorized users.

## Background

The numbers of patients with Alzheimer’s disease (AD) and type 2 diabetes mellitus (T2DM) both have been steadily increasing, imposing a serious public health problem in aging societies. AD is the most common neurodegenerative disorder characterized by the progressive decline in memory and cognitive functions. Deposition of amyloid-β peptides (Aβ) as senile plaques in the brain is the hallmark pathology of AD, and it is now generally believed that multimerization and accumulation of Aβ is a primary cause of neurodegeneration in AD brains [1]. In contrast to the rare, autosomal dominantly inherited early-onset AD that has demonstrated the pathogenic significance of Aβ, the vast majority of AD cases manifest as a sporadic form, where environmental and genetic factors may contribute to the pathophysiology of the disease. Among such factors, growing evidence suggests an association between T2DM and AD. A number of longitudinal epidemiological studies have shown that the incidence of AD is ~ 1.5- to 2-fold higher in patients with T2DM [2, 3].

T2DM is a complex metabolic disease characterized by defects in insulin action, progressive β-cell dysfunction and hyperglycemia. Although there are shared features between the pathophysiology underlying both diseases, the detailed molecular mechanism as to how T2DM increases the risk of AD remains to be solved [4]. Insulin resistance, the core component of T2DM, is developed in the early, pre-diabetes stage by a multifactorial process with environmental and genetic influences [5]. Recent studies have suggested that insulin resistance is also developed in the brains of AD patients, leading to the notion that insulin resistance is involved in the potential causal mechanism of AD [6–8].

Insulin resistance could influence the pathophysiology of AD in two possible manners. Insulin in the brain is thought to derive mainly from the periphery. In the central nervous system, insulin is involved in the regulation of the feeding behavior, whole body energy metabolism, as well as in memory and cognition by modulating synaptic function, neuronal survival, and neuronal glucose metabolism [9–11]. Patients with T2DM have been shown to develop cognitive dysfunction [12], in which dysregulation of brain insulin signaling is implicated. Recently, intranasal administration of insulin has shown to improve memory and cognitive functions in healthy elderly individuals as well as in patients with T2DM, mild cognitive impairment and AD [10, 13, 14]. Based on these observations, part of the cognitive deficits of AD due to T2DM could be attributed to the impaired brain insulin signaling [15].

The other important aspect connecting insulin resistance and the risk of AD is the effect on AD-related neuropathology in the brain. Peripheral insulin resistance was shown to be associated with a higher risk of AD [16]. Furthermore, a histological study on postmortem brains of a large cohort Hisayama study revealed that insulin resistance was associated with an increased risk of senile plaque formation [17]. The association between insulin resistance and Aβ accumulation in the brains of middle aged adults has also been shown by an amyloid PET neuroimaging study [18]. Studies using various experimental mouse models of AD support the observations in humans; induction of insulin resistance and related diabetic phenotypes by feeding a high-fat diet (HFD) increased Aβ levels and amyloid deposition in the brains of AD model mice [19–21]. Collectively, insulin resistance either in the periphery or in brains appears to be correlated with the Aβ accumulation.

A majority of previous studies in humans and research models suffered from limitations in unraveling the causal relations between the development of insulin resistance and the progression of amyloid pathology, because they have often been conducted in late stages of the disease where the AD pathology, especially the amyloid pathology has been fully developed. In considering the potential application of anti-diabetic therapeutic strategies on AD, it is critical to determine whether T2DM-induced exacerbation of AD pathology would be a reversible process. Furthermore, the molecular mechanisms of “brain insulin resistance”, which could either be due to impaired response of brain cells to the extracellular insulin or disrupted transport of blood insulin into the brain, have long remained unclear [22]. Notably, an in vivo examination of parenchymal insulin in relation to the metabolic abnormalities within brain areas affected in AD, e.g., hippocampus and cerebral cortex, has been lacking. It is also unknown how insulin resistance affects the brain Aβ dynamics in vivo. Another important question arose from the observations in mice that lack genes encoding components of the insulin signaling pathway, which have suggested the protective effects of genetically reduced insulin signaling on amyloid deposition, cognitive function and survival of AD model mice [23–26]. These lines of paradoxical observations raised a question as to the consequences of reduced insulin signaling on AD pathophysiology.

To address these unresolved problems, we have investigated APP transgenic mice in two independent insulin-resistant conditions, i.e., HFD feeding and genetic disruption of insulin receptor substrate 2 (IRS-2). In these models, we have examined the precise chronological changes of the development of peripheral/central insulin resistance in relation to the brain amyloid dynamics in vivo. We also aimed to determine the relationship between the insulin-resistant states and amyloid pathology, by combining *Irs2* deletion and dietary interventions with HFD feeding in the AD model mice. Our results indicated the causal and reversible effects of diet-induced, but not genetically-induced, insulin resistance on brain Aβ clearance and amyloid pathology. These observations raise the possibility that factors involved in the initiation of insulin resistance, e.g., metabolic stress induced by HFD-loading, but not impaired insulin signaling per se, might be directly involved in the induction of exacerbation of amyloid pathology in the brain.

## Methods

### Animals

A7 transgenic mice (A7-Tg) overexpressing human APP695 harboring KM670/671NL and T714I familial AD mutations under the control of the Thy-1.2 promoter [27] were backcrossed and maintained on a C57BL/6J background. *Irs2*^*−/−*^ mice were generated as previously described [28]. *Irs2*^*+/−*^ mice were crossed with A7-Tg mice to generate *Irs2*^*−/−*^;A7-Tg mice, and littermate controls were used. All experiments in this study were performed using male mice, unless otherwise stated. The animal care and experimental procedures were approved by the animal experiment committee of The University of Tokyo Graduate School of Medicine.

Mice were maintained on a 12 h light/dark cycle and provided ad libitum access to water. During the feeding protocols, mice were fed a standard chow diet (CRF-1, Oriental Yeast Co., Ltd.) until 3 months of age. Then they were either maintained on the standard chow or were switched to a high-fat diet (HFD) containing 32% fat (HFD32, CLEA Japan Inc.). For dietary intervention studies, groups of HFD-fed mice were switched back to the standard chow diet or caloric restriction (CR) at 9 months of age. A CR group was fed 70% of the food intake of the same genotype ad libitum controls.

### Metabolic measurements

Plasma concentrations of insulin were measured using a mouse insulin ELISA kit (Morinaga Institute of Biological Science, Inc.). Blood glucose was measured using Glutest sensor (Sanwa Kagaku Kenkyusho Co., LTD.). For an insulin tolerance test (ITT), overnight-fasted mice were intraperitoneally injected with human insulin (Humulin R, Eli Lilly) at 0.75 U/Kg body weight, and blood glucose was measured at different time points.

### Protein extraction and Western blot analysis

Brains were harvested, dissected into the hippocampus, cerebral cortex and hypothalamus, snap frozen in liquid nitrogen and stored at − 80 °C. For in vivo insulin stimulation experiments, 5-month-old mice were fasted for 4 h and intraperitoneally injected with 1 U of human insulin or phosphate-buffered saline (PBS) pH 7.4 as a control. For analysis at 9 and 15 months of age, mice were fasted overnight and intraperitoneally injected with 5 U of human insulin or PBS as a control. The mice were harvested after 40 min.

Brain tissues were homogenized in a 1:10 (w/v) volume of Tris-buffered saline (TBS), centrifuged at 260,000 x g for 20 min at 4 °C and supernatants were saved as TBS-soluble fractions. Resulting pellets were homogenized in a 1:10 (w/v) volume of 2% Triton X-100 in TBS and centrifuged at 260,000 x g for 20 min at 4 °C. Pellets were then homogenized in a 1:10 (w/v) volume of 2% SDS in TBS, incubated for 30 min at 37 °C and centrifuged at 260,000 x g for 20 min at 20 °C. SDS-insoluble pellets were dissolved in 70% formic acid using a sonicator (Branson), centrifuged at 260,000 x g for 20 min at 4 °C and supernatants were desiccated by Speed-Vac followed by resuspension in DMSO. Protein concentration was determined with BCA protein assay kit (ThermoFisher). All the buffers contained cOmplete protease inhibitor and PhosSTOP phosphatase inhibitor cocktails (Merck).

For detection of phosphorylation of the insulin receptor (IR), Triton X-100 soluble fractions were incubated with anti-insulin receptor β antibodies (C-19, Santa Cruz) overnight at 4 °C. Protein G agarose beads (ThermoFisher) were added and incubated for 2 h at 4 °C. The samples were washed three times with 2% Triton X-100 in TBS and eluted with Laemmli sample buffer containing 2% 2-mercaptoethanol.

For immunoblotting, samples were separated by SDS-polyacrylamide gel electrophoresis under a reducing condition using a Tris-Tricine gel system, transferred to polyvinylidene difluoride membrane (Merck) and reacted with antibodies. The immunoblots were developed using ImmunoStar reagents (Wako) and visualized by LAS-4000 mini (Fujifilm). The antibodies and dilutions used in this study were: anti-insulin receptor β C-19 (Santa Cruz) 1:2000, anti-phosphotyrosine 4G10 (Merck) 1:2000, anti-IDE ST1120 (Merck) 1:2000, anti-α-tubulin DM1A (Merck) 1:10000.

### Immunohistochemical analysis and morphometry

Mouse brains were fixed with 4% paraformaldehyde in PBS for 24 h, dehydrated and embedded in paraffin. Serial sections were cut at 4-μm thickness. Deparaffinized sections were treated with microwave (700 W) in citrate buffer pH 6.0 for 18 min, followed by digestion with 100 μg/ml proteinase K (Worthington) in TBS for 6 min at 37 °C. After blocking by incubation with 10% calf serum in TBS, the sections were incubated with an anti-Aβ antibody 82E1 (IBL) and then a biotinylated anti-mouse IgG antibody (Vector Laboratories), followed by visualization by avidin-biotin complex method (ABC elite, Vector Laboratories) using diaminobenzidine as chromogen. The sections were lightly counterstained with hematoxylin. The amyloid plaque burden of the indicated brain area was measured using Image J software (NIH) as previously described [29].

### In vivo microdialysis and glucose clamp

For the analyses of interstitial fluid (ISF) insulin, mice were anesthetized and infusion catheters were inserted into the right jugular vein 2–3 days before the glucose clamp experiment. At the same time, microdialysis guide cannulas (AtmosLM, Eicom) were stereotaxically implanted into the left hippocampus (from bregma: − 2.8 mm AP, 0.5 mm ML, − 1.3 mm DV at 38° angle) and fixed by dental cement. One day before clamps, AtmosLM probes with 1000 kDa molecular weight cut-off (MWCO) membranes (Eicom) were inserted into the guide cannula and mice were placed into Raturn cage system (BASi). Artificial cerebrospinal fluid (aCSF; 1.3 mM CaCl_2_, 1.2 mM MgSO_4_-7H_2_O, 3.0 mM KCl, 0.4 mM KH_2_PO_4_, 25 mM NaHCO_3_ and 122 mM NaCl) containing 0.15% bovine serum albumin (BSA, Sigma) was perfused at 0.1 μl/min. On the next day, insulin was continuously infused via the jugular line at the dose of 250 mU/kg/min for 10 min and 50 mU/kg/min for 230 min (240 min in total). The blood glucose concentration was monitored every 10 min during the first 60 min and every 15 min afterwards by Glutest sensor via tail-tip bleeds. Based on that, glucose infusion rate (GIR, mg/kg/min) was changed to maintain the blood glucose at approximately 60 mg/dl by the administration of 50% dextrose infusate (Otsuka) via the jugular line. The GIR was initially set at 60 and was adjusted accordingly. Blood samples were collected from the tail-tip at 0, 30, 60, 120, 180 and 240 min, plasma insulin concentrations were measured by ELISA, and the average plasma insulin levels were determined. ISF was pooled throughout the 4-h clamp in one tube and the levels of insulin in the pooled ISF samples were determined by ultra-sensitive PLUS mouse insulin ELISA kit (Morinaga Institute of Biological Science, Inc.).

For the analyses of ISF Aβ, microdialysis guide cannulas (BASi) were implanted into the left hippocampus. Microdialysis probes with 30 kDa MWCO membranes (BASi) were inserted and aCSF containing 0.15% BSA was perfused at 1 μl/min. ISF samples were collected every 1 h. To determine the half-life of ISF Aβ, compound E (Merck) was infused via reverse microdialysis for 6 h at 1 μM after overnight collection of the baseline ISF. Aβ half-life was determined as described previously [30].

### ELISA quantitation of Aβ

To measure the Aβ levels in ISF and brain soluble fractions, an equal volume of 1 M guanidine hydrochloride was added and incubated for 30 min at room temperature. Levels of Aβ were quantitated by BNT77/BA27 or BNT77/BC05 Human/Rat β Amyloid ELISA kit (Wako) [27].

### Acute brain slices

Brains were removed and rapidly placed into chilled aCSF. The brain was cut by a razor blade coronally to remove the forebrain, brain stem and the cerebellum. The block was glued onto the vibratome stage and covered with chilled aCSF. Acute brain slices containing the hippocampus and cortex were made at 300 μm intervals. During the cut, aCSF was bubbled with 95% O_2_/5% CO_2_. Each slice was transferred on a cell strainer with a 100-μm pore size mesh (ThermoFisher) within a chamber containing a medium (25% inactivated horse serum, 0.5× MEM, 0.25× Hank’s BSS, 5 mM Tris and 26 mM sodium bicarbonate) and placed in a tissue culture incubator. Cultured slices were homogenized in an equal amount of RIPA buffer (1% NP-40, 0.5% sodium deoxycholate, 0.1% SDS in TBS) containing cOmplete protease inhibitor cocktail and centrifuged at 260,000 x g for 20 min at 4 °C. Aβ levels in the culture media were measured using ELISA and adjusted by the protein content of each slice.

### Analysis of NEP activity

Brain tissues were homogenized in a 1:10 (w/v) volume of TBS and centrifuged at 260,000 x g for 20 min. The pellet was resuspended with 50 mM Tris-HCl pH 7.6 by passing through a 27-gauge syringe needle. Protein concentration was determined with BCA protein assay. The lysates were incubated with 50 μM 3-dansyl-D-Ala-Gly-p-nitro-Phe-Gly (DAGNPG, Merck) and 1 μM captopril in 50 mM Tris-HCl pH 7.6 at 37 °C. After 2 h incubation, reactions were stopped by heating for 5 min at 100 °C and the samples were centrifuged at 1000 x g for 5 min. The supernatant was diluted with 50 mM Tris-HCl pH 7.6 and the fluorescence signals were measured using SpectraMax M2 (Molecular Devices) with the settings: excitation 342 nm; emission 562 nm.

### Quantitative reverse transcription PCR

Total RNA was isolated using ISOGEN reagent (Nippon Gene). RNA purity and concentration were measured by Nanodrop (ThermoFisher). Total RNA was reverse-transcribed into cDNA using ReverTra Ace qPCR RT Master Mix with gDNA Remover (TOYOBO). Real-time PCR was performed using THUNDERBIRD SYBR qPCR Mix (TOYOBO) in LightCycler 450 system (Roche) in 384-well plates. Threshold cycle values were normalized to Gapdh. Primer pairs used in this study are as follows: 5′-AACGACCCCTTCATTGAC-3′ and 5′-GAAGACACCAGTAGACTCCAC-3′ for Gapdh; 5′-GATTATGGCTCAGGGTCCAA-3′ and 5′-GATTATGGCTCAGGGTCCAA-3′ for TNFα; 5′-AGTGGTGGCCACTAATGGAG-3′ and 5′-CAATCCTTGCTTGATGCTGA-3′ for Grp78/BiP; 5′-CCTAGCTTGGCTGACAGAGG-3′ and 5′-CTGCTCCTTCTCCTTCATGC-3′ for CHOP.

### Statistical analysis

Quantitative data were analyzed statistically by two-tailed, unpaired t test for two-group data, one-way with repeated measures for data with multiple time points, or one-way or two-way ANOVA followed by post-hoc tests (as appropriate) for multiple group comparisons using GraphPad Prism 6. In figures, all data are represented by mean ± SEM. Statistical significance is indicated by *p < 0.05, ** *p* < 0.01, and *** *p* < 0.001.

## Results

### High-fat diet feeding led to obesity and peripheral insulin resistance in A7-Tg mice

**Fig. 1:**
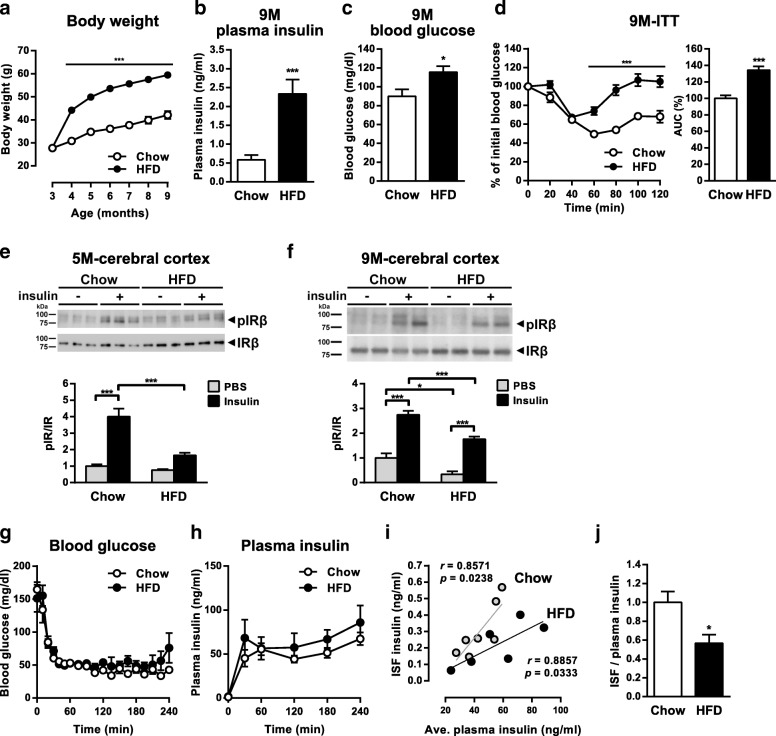
Long-term HFD feeding induces peripheral and brain insulin resistance in A7-Tg mice. **a** Monthly body weight changes of male A7-Tg mice (Chow: *n* = 12; HFD: *n* = 11). **b,c** Fasting plasma insulin (**b**) and blood glucose levels (**c**) of 9-month-old male A7-Tg mice (Chow: *n* = 12; HFD: *n* = 11). **d** Blood glucose levels during an insulin tolerance test (ITT, 0.75 U/kg body weight, left) and the area under the curve (AUC) of blood glucose (right) of 9-month-old male A7-Tg mice (Chow: *n* = 12; HFD: *n* = 10). **e, f** Levels of phosphorylated IR in the brain upon insulin administration. A7-Tg mice were intraperitoneally injected with PBS or insulin and cortical lysates were immunoprecipitaed with an anti-IR antibody followed by immunoblotting with anti-phospho Tyr and anti-IR antibodies at 5 (**e**, Chow-PBS: *n* = 3; Chow-insulin: *n* = 6; HFD-PBS: *n* = 4; HFD-insulin: *n* = 6) and 9 (**f**, Chow-PBS: *n* = 6; Chow-insulin: *n* = 6; HFD-PBS: *n* = 5; HFD-insulin: *n* = 6) month of age (upper panels). Relative levels of signal intensity are shown (lower panels). **g** Blood glucose levels during hyperinsulinemic glucose clamps (Chow: *n* = 7; HFD: *n* = 6). **h** Plasma insulin levels during the clamps (Chow: *n* = 7; HFD: *n* = 6). **i** Correlation plots between the ISF insulin and the average plasma insulin levels during the clamps in chow and HFD-fed A7-Tg mice (Chow: *n* = 7; HFD: *n* = 6). **j** ISF to plasma insulin ratio (Chow: *n* = 7; HFD: *n* = 6). Data are mean ± SEM. *p < 0.05, **p < 0.01, *** *p* < 0.001 (repeated-measures ANOVA with Sidak’s post-hoc test, **a, d**; unpaired *t* test, **b, c, d, j**; two-way ANOVA with Tukey’s post-hoc test, **e, f**; Spearman’s correlation, **i**)

Transgenic mice used in this study overexpress human APP harboring the Swedish and Austrian mutations under the control of the Thy-1.2 promoter (A7-Tg; [27]). A7-Tg mice develop progressive Aβ deposition in brains starting at ~ 12 months of age. In this study, we focused on three different age ranges with distinct pathological stages of A7-Tg mice: 5 months of age when brain Aβ is almost soluble, 9 months when the levels of both soluble and insoluble Aβ become gradually elevated with no visible amyloid plaques, and 15–18 months when amyloid plaques are massively accumulated. To investigate the effect of diet-induced obesity and insulin resistance on Aβ pathology, we fed A7-Tg mice with a HFD starting from 3 months of age (Additional file 1: Figure S1a). At 3 months of age, no difference was found in the peripheral insulin sensitivity between A7-Tg and wild-type mice (Additional file 1: Figure S1b). After 6 months of treatment, 9-month-old HFD-fed male A7-Tg mice showed increases in body weight, fasting plasma insulin, fasting blood glucose, and reduced insulin sensitivity as measured by an insulin tolerance test (Fig. 1a-d). We also confirmed the effects of HFD feeding in female A7-Tg mice, which showed insulin resistance (Additional file 1: Figure S1c-g). These data suggest that ad libitum intake of the HFD induced metabolic dysregulation in A7-Tg mice.

### Impaired brain insulin response in HFD-fed A7-Tg mice

Previous studies on animal models of diet-induced obesity have provided evidence that insulin resistance is induced in the hypothalamus, resulting in impaired regulation of whole-body energy expenditure [31]. However, it is not clear whether insulin resistance takes place in the whole brain. We investigated the relationship between HFD-induced Aβ pathology and insulin signaling in the cerebral neocortices, where AD pathology is typically present in humans and model mice. Peripherally administered insulin stimulated tyrosine phosphorylation of the insulin receptor (IR) β-subunit in the cerebral cortices of A7-Tg mice (Fig. 1e). Intraperitoneal injection of 1 U of insulin in 5-month-old A7-Tg mice showed that insulin-dependent phosphorylation of IR in the brain was significantly suppressed by HFD feeding (Fig. 1e). Nine-month-old A7-Tg mice were also examined by injecting 5 U of insulin, in which the levels of elevated plasma insulin were comparable between control and HFD mice (11.58 ± 1.75 vs 10.51 ± 1.29 μg/ml, not significant). When the extent of phospho-IR was compared between PBS- and insulin-treated groups, HFD-fed A7-Tg mice showed a higher rate of change compared with the control mice (Fig. 1f). This might be due to the differential effect of HFD feeding on the basal levels, and acute response to insulin, of phosphorylation of IR in the brain. However, the absolute levels of both steady state and insulin-induced phospho-IR were significantly reduced in the HFD group compared with the control (Fig. 1f). The levels of insulin-dependent tyrosine phosphorylation of IGF-1R and IRS-2 were also lower in HFD-fed A7 brains, although the difference between chow and HFD-fed groups was not statistically significant (Additional file 1: Figure S1 h and i). Altogether, these observations suggest that brain response to peripheral insulin stimulation was impaired at as early as 5 months of age.

### Reduced ISF/plasma insulin ratio during the hyperinsulinemic glucose clamp in HFD-fed A7-Tg mice

Although the mechanisms underlying the brain insulin resistance are not fully elucidated, it is speculated to result either from impaired response in neuronal insulin signaling or reduced blood-to-brain transport of insulin, or both [22]. Our in vivo insulin stimulation analysis demonstrated reduced kinase activation of IR, an immediate target of insulin, in the brains of HFD-fed A7-Tg mice, which led us to examine if the levels of brain interstitial insulin are reduced. To directly assess the effect of HFD-induced metabolic abnormalities on delivery of insulin across the vascular barrier and central insulin levels, we performed hyperinsulinemic glucose clamps combined with in vivo brain microdialysis using 1000 kDa molecular weight cut-off (MWCO) probes [32]. Insulin was infused via the jugular line, while blood glucose levels were maintained constant by administrating dextrose through the jugular line (Fig. 1g). During the 4-h clamps, plasma insulin levels were elevated and not different between the control and HFD-fed groups (Fig. 1h). We then measured the insulin levels in the brain interstitial fluid (ISF), which were 0.304 ± 0.061 and 0.220 ± 0.055 ng/ml in chow and HFD-fed A7-Tg mice, respectively. The levels of basal ISF insulin were not detectable in our study. Although the levels of ISF insulin during the clamp were considerably lower than that of plasma insulin, these were positively and significantly correlated with the average concentrations of plasma insulin both in chow-fed (*r* = 0.8571, *p* = 0.0238) and HFD-fed (*r* = 0.8857, *p* = 0.0333) A7-Tg mice (Fig. 1i). When the ratios of ISF insulin to the average plasma insulin levels were compared between the control and HFD groups, HFD-fed A7-Tg mice showed a significantly lower ISF/plasma ratio (Fig. 1j). These results raised the possibility that the transport of exogenously administrated insulin to the brain was impaired by HFD feeding in A7-Tg mice.

### Insulin resistance precedes HFD-induced exacerbation of Aβ deposition in the brains of A7-Tg mice

**Fig. 2:**
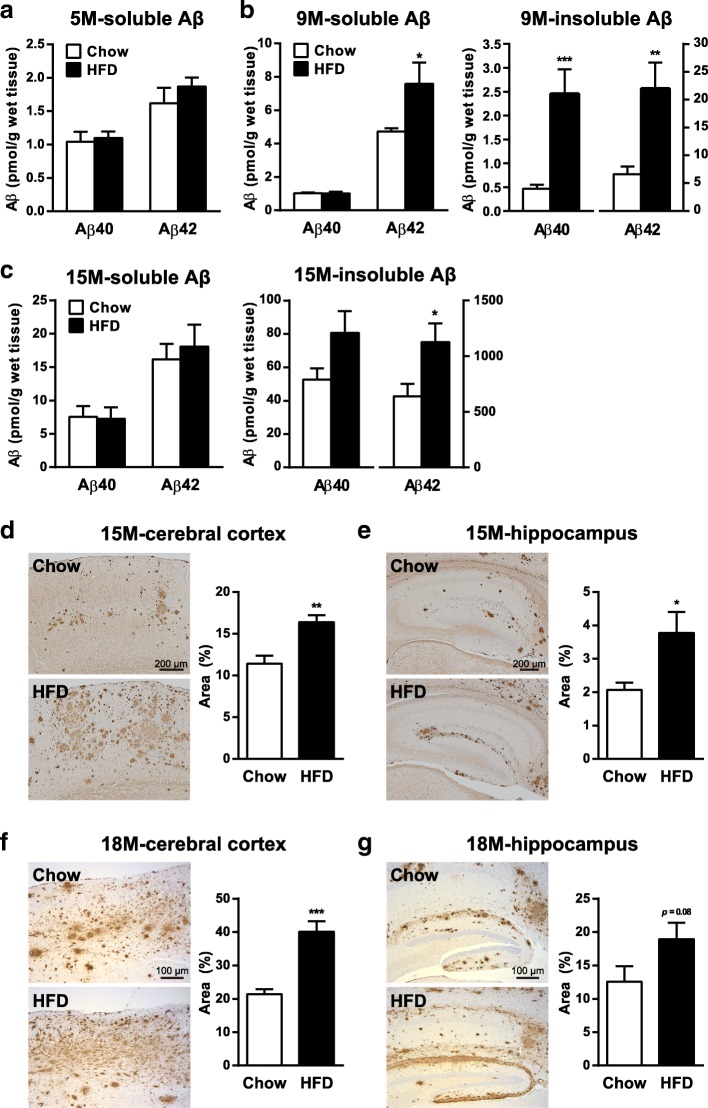
Development of insulin resistance precedes HFD-induced exacerbation of Aβ deposition in A7-Tg mouse brains. **a** Soluble Aβ40 and Aβ42 levels in the cerebral cortex of 5-month-old male A7-Tg mice fed with chow (*n* = 9) or HFD (*n* = 10). **b** Soluble (left) and insoluble (right) Aβ40 and Aβ42 levels in the cerebral cortex of 9-month-old male A7-Tg mice fed with chow (*n* = 12) or HFD (*n* = 11). **c** Soluble (left) and insoluble (right) Aβ40 and Aβ42 levels in the cerebral cortex of 15-month-old female A7-Tg mice fed with chow (*n* = 5) or HFD (*n* = 6). **d-g** Immunohistochemical analysis of female A7-Tg mouse brains using an anti-Aβ (82E1) antibody. Representative images of the cerebral neocortex at the level of hippocampus (**d**), hippocampus (**e**) and the percentage area covered by Aβ immunoreactivity (right panels in each figure) from 15-month-old A7-Tg mice fed with chow (*n* = 5) or HFD (*n* = 6). Data from the cerebral neocortex (**f**) and hippocampus (**g**) of 18-month old A7-Tg mice fed with chow (*n* = 9) or HFD (*n* = 7) also are shown. Data are mean ± SEM. *p < 0.05, ** *p* < 0.01, *** *p* < 0.001 (unpaired *t* test)

To investigate the relationship between the HFD-induced impairment in insulin signaling and the amyloid pathology, we examined the longitudinal changes in Aβ pathology in the brains of HFD-fed A7-Tg mice. First, we biochemically investigated the chronological relation between the HFD-induced impaired insulin signaling and the Aβ deposition. Two-month feeding on the HFD, which significantly suppressed insulin responses in brains (Fig. 1e), had no effect on the levels of Aβ40 and Aβ42 in TBS-soluble extracts of the cerebral cortices of 5-month-old A7-Tg mice yet (Fig. 2a). At 9 months of age, i.e., prior to plaque deposition, HFD feeding already led to an increase in cerebral Aβ40 and Aβ42 levels both in TBS-soluble and TBS/Triton X-100/SDS-insoluble fractions (Fig. 2b). There also was a significant increase in the levels of Aβ42 in the hippocampus of HFD-fed A7-Tg mice, where amyloid deposition occurs slightly later than in cortices in A7-Tg mice (Additional file 2: Figure S2). At 15 months of age, the concentrations of both soluble and insoluble Aβ in the brain were increased in proportion to the deposition of amyloid plaques, and HFD feeding further increased the levels of insoluble Aβ42 in the neocortices of A7-Tg mice (Fig. 2c).

Consistent with earlier studies in other AD mouse models [19–21], Aβ deposition was significantly enhanced by HFD feeding in the neocortices as well as hippocampus of aged A7-Tg mice (Fig. 2d-g). Quantification of percentage areas covered by Aβ immunoreactivity showed a ~ 2-fold increase in amyloid deposition in HFD-fed mice. These results revealed that the HFD-induced impairments in insulin signaling both in peripheral tissues and brains precede the increase in Aβ levels in the brains of A7-Tg mice.

### Decreased clearance of ISF Aβ in HFD-fed A7-Tg mice

**Fig. 3:**
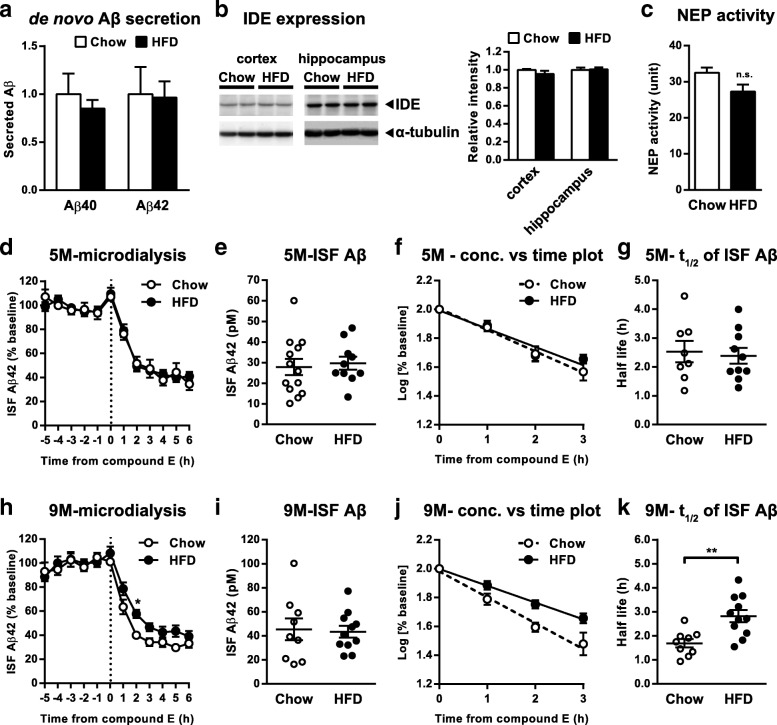
Chronic HFD feeding affects the clearance of ISF Aβ. **a** Secreted Aβ40 and Aβ42 from acute brain slices of 10-month-old A7-Tg mice fed with chow (*n* = 8) or HFD (*n* = 9). **b** Immunoblot and densitometric analyses of IDE protein in the neocortical and hippocampal lysates of 9**-**month-old A7-Tg mice fed with chow (*n* = 12) or HFD (*n* = 11). **c** NEP activities in microsomal fractions from the cerebral neocortices of 9-month-old A7-Tg mice fed with chow (*n* = 6) or HFD (*n* = 5). **d-k** The levels and half-life of ISF Aβ42 were analyzed by microdialysis. **d, h** Hourly measurements of ISF Aβ42 during microdialysis in 5 (**d**) and 9 (**h**)-month-old A7-Tg mice fed with chow (**d**: *n* = 8; **h**: *n* = 9) or HFD (**d**: *n* = 10; **h**: *n* = 11). ISF Aβ42 levels are shown as % of mean baseline of chow-fed mice. **e, i** Baseline levels of ISF Aβ42 during the 6-h sampling period before compound E treatment in 5 (**e**) and 9 (**i**)-month-old A7-Tg mice fed with chow (**e**: *n* = 13; **i**: *n* = 9) or HFD (**e**: *n* = 10; **i**: *n* = 11). **f, j** Rate of reduction in ISF Aβ42 after administration of compound E in 5 (**f**) and 9 (**j**)-month-old A7-Tg mice. A semi-log plot of percentage ISF Aβ42 levels (2.0 at time 0) is shown versus time. **g, k** Half-life of ISF Aβ42 in 5 (**g**) and 9 (**k**)-month-old A7-Tg mice fed with chow (**g**: *n* = 8; **k**: *n* = 9) or HFD (**g**: *n* = 10; **k**: *n* = 11) based on data in **f** or **j**. Data are mean ± SEM. ** *p* < 0.01 (unpaired *t* test, **a**-**c**, **e**, **g**, **i**, **k**; repeated-measures ANOVA with Sidak’s post-hoc test, **d, h**)

The levels of Aβ in the brain are regulated by a balance between production, clearance and aggregation. We therefore examined which step(s) of Aβ dynamics may account for the exacerbation of amyloid pathology in the HFD-fed A7-Tg brains. Immunoblot analysis of APP, C-terminal fragments (CTFs) of APP, β-secretase (BACE1) and α-secretase (ADAM10) showed no significant differences between the cerebral cortex of 5-month-old control and HFD-fed A7-Tg mice (Additional file 3: Figure S3a). At 9 months of age, the levels of CTFα and CTFβ in HFD-fed mice were slightly higher than that of the control group (Additional file 3: Figure S3b), which could be due to either altered APP processing by α- and β-secretases or decreased clearance of the APP fragments. To evaluate the effects of HFD on amyloidogenic APP processing, we measured the amount of Aβ released from acute brain slices of A7-Tg mice. Levels of Aβ40 and Aβ42 in the media of 10-month-old HFD-fed A7-Tg brain slice were not different from that of control brain slice (Fig. 3a), and neither of the levels of total sAPP, sAPPα nor sAPPβ were altered in the media of HFD-fed mouse slice cultures (Additional file 3: Figure S3c). These results suggest that increased Aβ production may not be responsible for the incremental effect of HFD on Aβ deposition.

Several studies have shown that HFD feeding affects the expression levels and/or activities of Aβ degrading enzymes, i.e., insulin degrading enzyme (IDE) and neprilysin (NEP) [19, 33, 34]. In our model, however, we did not find any effects of HFD feeding on IDE levels or neprilysin activity in the brains of 9-month-old A7-Tg mice (Fig. 3b and c).

Since no in vivo study has so far investigated the effect of diet-induced metabolic abnormalities on the brain Aβ metabolism, we examined the levels and half-life (*t*_*1/2*_) of Aβ in the brain ISF by using in vivo microdialysis technique. To measure ISF Aβ, mice were implanted with microdialysis probes with 30 kDa MWCO in the hippocampus and ISF was collected every 60 min [30]. Baseline levels of ISF Aβ42 were similar between chow and HFD-fed A7-Tg mice at 5 months of age (Fig. 3d and e). The *t*_*1/2*_ of ISF Aβ42 was then determined by administering a potent γ-secretase inhibitor compound E locally through reverse dialysis. Following compound E infusion, ISF Aβ42 levels were rapidly decreased by ~ 60% compared to the baseline (Fig. 3d). A semi-log plot of concentration versus time suggested first-order kinetics for ISF Aβ42 (Fig. 3f). The *t*_*1/2*_ calculated based on the slope of decline in the levels of ISF Aβ42 was comparable between the control and HFD-fed groups at 5 months of age (2.52 ± 0.370 h and 2.38 ± 0.274 h, respectively, Fig. 3g). In 9-month-old A7-Tg mice, baseline levels of ISF Aβ42 were also not significantly different between the control and HFD-fed A7-Tg mice (Fig. 3h and i). Interestingly, however, elimination of ISF Aβ42 after compound E treatment was markedly delayed by HFD feeding. Analysis of the *t*_*1/2*_ for ISF Aβ42 was 1.69 ± 0.176 h in the control mice and 2.82 ± 0.254 h in HFD-fed mice, showing a significant difference between the two groups (*p* = 0.0026, Fig. 3j and k). Together, these observations suggest that the reduced clearance of Aβ might be involved in the HFD-induced exacerbation of Aβ pathology.

### Dietary interventions reverse HFD-induced metabolic impairments and accelerated Aβ pathology in A7-Tg mice

Next, we aimed to examine if an improvement in insulin resistance could reverse the exacerbating effects of HFD on Aβ pathology. To test this, we employed dietary interventions, which have been shown to rescue metabolic deficiencies caused by the HFD [35]. In the A7-Tg mouse model, we first verified beneficial effects of 30% caloric restriction (CR) on the metabolic parameters. CR suppressed body weight gain, and increased both peripheral and central insulin sensitivity (Additional file 4: Figure S4a-d).

**Fig. 4:**
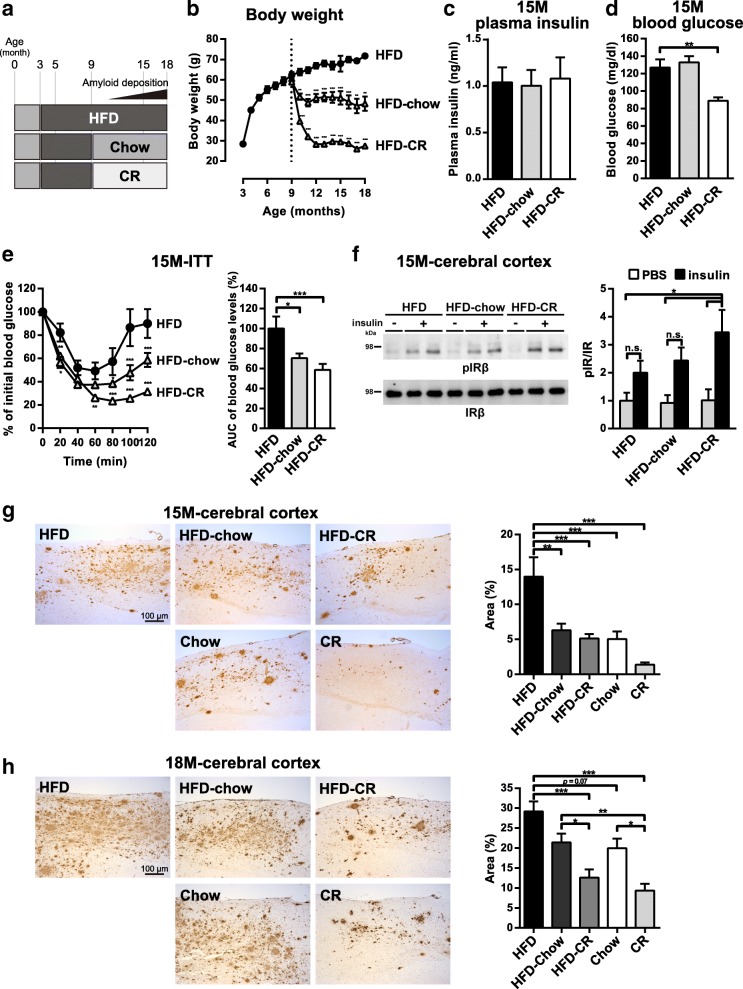
Dietary interventions ameliorate HFD-induced metabolic impairments and Aβ pathology in A7-Tg mice. **a** Experimental scheme. Male A7-Tg mice were fed with HFD for 6 months, followed either by the chow diet (HFD-Chow), 30% calorie-restricted diet (HFD-CR) or remained on HFD for 9 months. **b** Monthly body weight changes of A7-Tg mice fed with HFD-Chow, HFD-CR or HFD (*n* = 12 per group). **c** Fasting plasma insulin levels of 15-month-old A7-Tg mice fed with HFD (*n* = 10), HFD-Chow (*n* = 11) or HFD-CR (*n* = 11). **d** Fasting blood glucose levels of 15-month-old A7-Tg mice fed with HFD (*n* = 8), HFD-Chow (*n* = 10) or HFD-CR (*n* = 11). **e** Blood glucose levels of 15-month-old A7-Tg mice fed with HFD (*n* = 8), HFD-Chow (*n* = 9) or HFD-CR (*n* = 11) during the ITT (left) and the AUC of blood glucose (right). **f** Immunoblot (left) and densitometric (right) analyses of phosphorylated/total IR levels in the cortices of 15-month-old A7-Tg mice fed with HFD (*n* = 6 and 4), HFD-Chow (*n* = 6 and 5) or HFD-CR (*n* = 5 and 6) treated with PBS or insulin. **g-h** Immunohistochemistry of Aβ deposition (left) and morphometry of percentage Aβ-positive areas (right) in the cerebral neocortices of 15 (**f**, HFD: *n* = 10; HFD-Chow: *n* = 10; HFD-CR: *n* = 11; Chow: *n* = 10; CR: *n* = 11) and 18 (**g**, HFD: *n* = 7; HFD-Chow: *n* = 12; HFD-CR: *n* = 12; Chow: *n* = 9; CR: *n* = 10)-month-old A7-Tg mice. Data are mean ± SEM. *p < 0.05, ** *p* < 0.01, *** *p* < 0.001 (repeated-measures ANOVA with Sidak’s post-hoc test, **b, e**; one-way ANOVA with Dunnett’s post-hoc test, **c, d, e**; two-way ANOVA with Tukey’s post-hoc test, **f**; one-way ANOVA with Tukey’s post-hoc test, **g, h**)

In our experimental settings, the effect of HFD-induced metabolic dysregulation on amyloid pathology was evident by 9 months of age. Thus, we switched the feeding of 9-months-old HFD-fed A7-Tg male mice to a regular chow or a regular chow with 30% CR (referred to as HFD-Chow or HFD-CR, respectively, Fig. 4a). After switching to the chow diet or CR, body weight was rapidly decreased within 2–3 months and remained stable afterwards (Fig. 4b). At the end of the experiments, the average body weight was very similar between HFD-Chow (48.2 ± 3.3 g, Fig. 4b) and the chow-fed control mice (44.6 ± 3.5 g, Additional file 4: Figure S4a), or between HFD-CR (27.4 ± 0.2 g, Fig. 4b) and CR mice (26.5 ± 0.3 g, Additional file 4: Figure S4a). Although the levels of fasting plasma insulin were not altered between HFD-Chow, HFD-CR and HFD groups (Fig. 4c), fasting blood glucose levels were significantly lower in 15-month-old HFD-CR mice (Fig. 4d) below those observed in CR and the chow control mice (Additional file 4: Figure S4b). The insulin tolerance test at 15 months of age demonstrated that HFD-induced insulin resistance was improved in both HFD-Chow and HFD-CR mice (Fig. 4e). The changes in blood glucose levels during the tests in HFD-Chow (~ 60% reduction at 60 min) and HFD-CR mice (~ 75% reduction at 60 min, Fig. 4e) were similar to those of mice continuously fed with each diet (Additional file 4: Figure S4c). We also examined brain insulin response to peripherally administered insulin. HFD-CR mice showed significantly increased IR phosphorylation in response to insulin, whereas HFD-Chow mice showed slightly improved response that was not significant compared to that of HFD-fed A7-Tg mice (Fig. 4f). These results indicated that dietary interventions improved both peripheral and central insulin signaling corresponding to the degree of diet restriction.

We next examined whether the rescue of metabolic abnormalities was associated with a reversal of HFD-induced exacerbation of amyloid pathology in A7-Tg mice. Immunohistochemical analysis showed that CR dramatically attenuated Aβ deposition in the brains of A7-Tg mice (Fig. 4g and h), consistent with the previously reported results in other APP transgenic mouse models [36, 37]. Notably, the dietary interventions significantly ameliorated Aβ pathology in the cerebral cortices of 15-month-old A7-Tg mice to the same extent as the chow-fed control mice (Fig. 4g). The protective effect of dietary intervention was also observed in the brains of 18-month-old HFD-CR mice (Fig. 4h). These results demonstrated that controlling diet can reverse the deteriorating effect of HFD on brain amyloid pathology even after HFD-induced elevation of Aβ levels has been initiated. Altogether, changing the states of the peripheral and brain insulin signaling in either way was correlatively associated with the brain Aβ metabolism.

### Deletion of IRS-2 induces metabolic impairments and suppresses Aβ accumulation in A7-Tg mice

Correlation between diet-induced metabolic phenotypes and modulation of Aβ pathology in A7-Tg mice raised a question as to what pathophysiological factor(s) of the metabolic syndrome, e.g. hyperinsulinemia, hyperglycemia, insulin resistance, are responsible for the induction of amyloid pathology. To address this issue, we next employed another diabetic mouse model that genetically lacks IRS-2. The mice deficient in IRS-2 have been shown to display T2DM-like phenotypes due to insulin resistance of the liver and defective pancreatic β-cell function [28]. Previous studies using Tg2576 APP transgenic mice crossed with *Irs2* knockout mice suggested a protective effect of IRS-2 disruption on Aβ pathology [23, 24]. To further examine the underlying mechanisms, we generated *Irs2*^*−/−*^;A7-Tg mice.

**Fig. 5:**
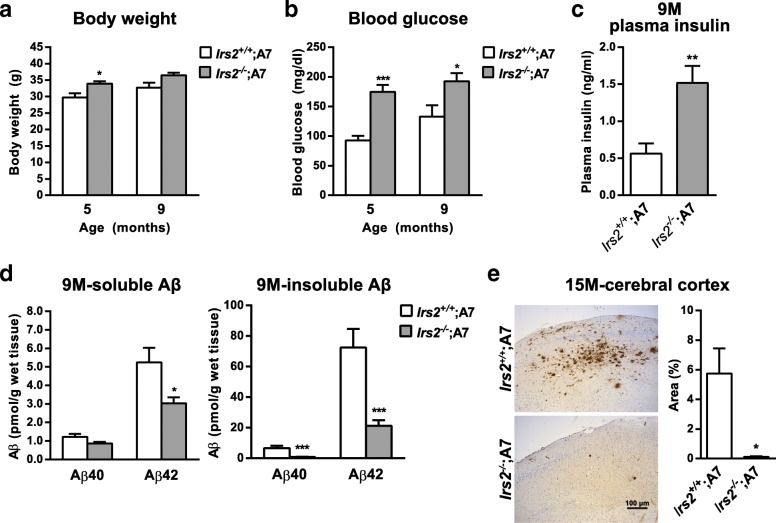
Deletion of IRS-2 induces metabolic impairments but suppresses Aβ deposition in A7-Tg mice. **a** Body weight of IRS-2-deficient A7-Tg mice at 5 and 9 month of age (*Irs2*^*+/+*^;A7-Tg mice: *n* = 6; *Irs2*^*−/−*^;A7-Tg mice: *n* = 5). **b** Fasting blood glucose levels of 5 and 9-month-old IRS-2-deficient A7-Tg mice (*Irs2*^*+/+*^;A7-Tg mice: *n* = 6; *Irs2*^*−/−*^;A7-Tg mice: *n* = 5). **c** Fasting plasma insulin levels of 9-month-old IRS-2-deficient A7-Tg mice (*Irs2*^*+/+*^;A7-Tg mice: *n* = 6; *Irs2*^*−/−*^;A7-Tg mice: *n* = 5). **d** Soluble (left) and insoluble (right) Aβ40 and Aβ42 levels in the cerebral neocortices of 9-month-old IRS-2-deficient A7-Tg mice (*Irs2*^*+/+*^;A7-Tg mice: *n* = 12; *Irs2*^*−/−*^;A7-Tg mice: *n* = 14). **e** Immunohistochemical analysis (left) and morphometry of percentage Aβ-positive areas (right) of cerebral neocortices of 15-month-old IRS-2-deficient A7-Tg mice (*Irs2*^*+/+*^;A7-Tg mice: *n* = 3; *Irs2*^*−/−*^;A7-Tg mice: *n* = 4). Data are mean ± SEM. *p < 0.05, *** *p* < 0.001 (unpaired *t* test)

*Irs2*^*−/−*^;A7-Tg mice displayed a slight increase in body weight and elevated fasting blood glucose at 5 and 9 months of age, indicating development of T2DM-like metabolic defects (Fig. 5a and b). The levels of fasting plasma insulin were also higher in 9-month-old *Irs2*^*−/−*^;A7-Tg mice (Fig. 5c). Biochemical analysis showed significantly lower levels of Aβ40 and Aβ42 both in soluble and insoluble cerebral fractions of 9-month-old *Irs2*^*−/−*^;A7-Tg mice compared to those of *Irs2*^*+/+*^;A7-Tg mice (Fig. 5d). In aged 15-month-old A7-Tg mice, the lack of IRS-2 significantly suppressed amyloid deposition in the cerebral cortex (Fig. 5e). These observations were in agreement with previous studies in which genetically induced impairment of insulin signaling significantly suppressed Aβ deposition in Alzheimer model mice [23, 24].

Given that the physiological consequences of increases in body weight, blood glucose, blood insulin and reduced insulin signaling on amyloid deposition were opposite between the models of HFD-induced insulin resistance and genetic disruption of the insulin signaling pathway, we sought to clarify the difference between the two diabetic models. To this end, we investigated the systemic inflammation and the endoplasmic reticulum (ER) stress, which have been implicated in the development of insulin resistance and T2DM [38]. mRNA levels of inflammatory cytokine TNFα and ER stress-related genes such as Grp78/BiP and CHOP in adipose tissue were significantly elevated by HFD feeding in *Irs2*^*+/+*^;A7-Tg mice, as expected (Additional file 5: Figure S5). In contrast, adipose tissue of *Irs2*^*−/−*^;A7-Tg did not show elevated expression of the genes despite the peripheral insulin resistance and diabetic phenotypes (Additional file 5: Figure S5). These results indicated that diet-induced obesity and metabolic stress might be one of the exacerbating factors of AD-like pathology.

### HFD feeding on IRS-2-deficient A7-Tg mice exacerbated diabetic phenotype as well as Aβ pathology

**Fig. 6:**
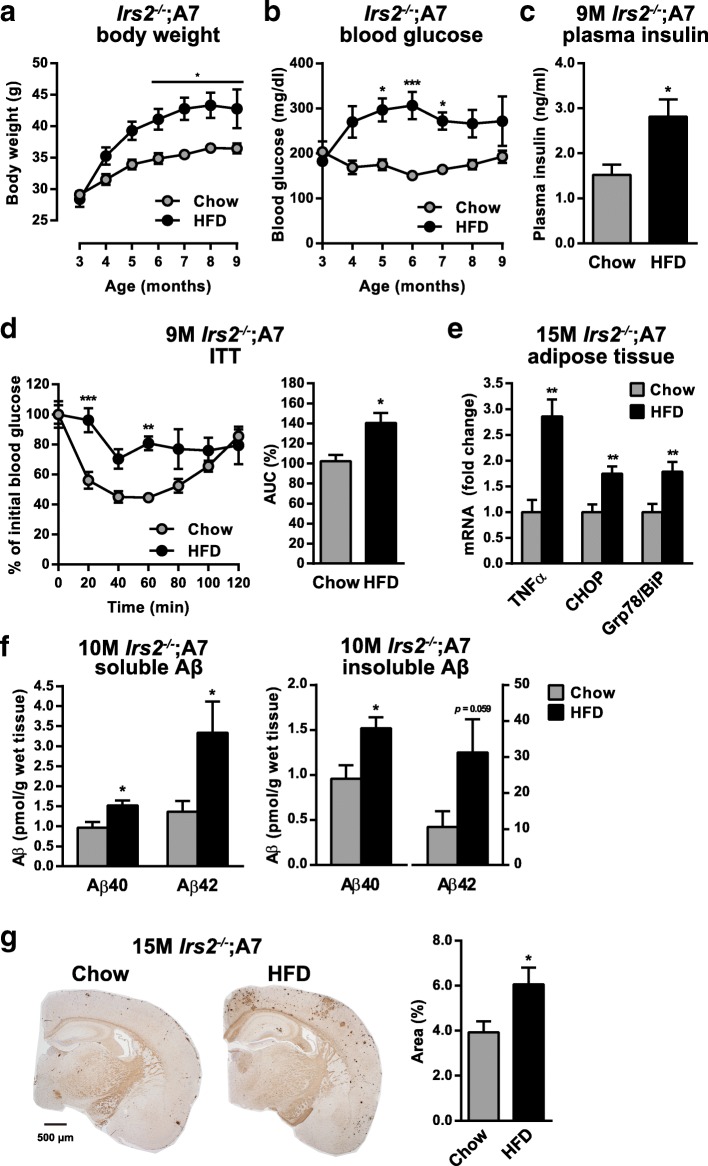
HFD feeding on IRS-2-deficient A7-Tg mice exacerbates diabetic phenotype and Aβ pathology. **a** Effect of HFD feeding on body weight of male *Irs2*^*−/−*^;A7-Tg mice (Chow: *n* = 5; HFD: *n* = 6). **b** Effect of HFD feeding on blood glucose levels of male *Irs2*^*−/−*^;A7-Tg mice (Chow: *n* = 5; HFD: *n* = 6). **c** Effect of HFD feeding on fasting plasma insulin levels of 9-month-old male *Irs2*^*−/−*^;A7-Tg mice (Chow: *n* = 5; HFD: *n* = 6). **d** Blood glucose levels during the ITT (left) and the AUC of blood glucose (right) at 9 months of age (Chow: *n* = 5; HFD: *n* = 3). **e** Quantitative RT-PCR analysis of TNFα, CHOP and Grp78/BiP mRNA expression in the adipose tissues of 15-month-old HFD-fed female *Irs2*^*−/−*^;A7-Tg mice (*n* = 6 per group). **f** Soluble (left) and insoluble (right) Aβ40 and Aβ42 levels in the cerebral cortices of HFD-fed male *Irs2*^*−/−*^;A7 mice at 10 months of age (Chow: *n* = 6; HFD: *n* = 5). **g** Immunohistochemical analysis (left) and morphometry of percentage Aβ-positive areas (right) of the cerebral neocortices of HFD-fed female *Irs2*^*−/−*^;A7 mice at 15 months of age (*n* = 6 per group). Data are mean ± SEM. *p < 0.05, **p < 0.01, *** *p* < 0.001 (repeated-measures ANOVA with Sidak’s post-hoc test, **a, b, d**; unpaired *t* test, **c-g**)

Based on the results from the two diabetic AD mouse models, we further examined the association between impaired insulin signaling, metabolic stress and Aβ pathology by feeding *Irs2*^*−/−*^;A7-Tg mice with HFD. During the 6 months of HFD feeding, male *Irs2*^*−/−*^;A7-Tg mice gained more weight than chow-fed controls (Fig. 6a). These mice also exhibited chronically higher levels of blood glucose (Fig. 6b). After 6 months of HFD feeding, 9-month-old *Irs2*^*−/−*^;A7-Tg mice exhibited increases in plasma insulin levels and insulin resistance, indicating the HFD-induced aggravation of metabolic abnormalities (Fig. 6c and d). Twelve months of HFD feeding caused a significant increase in body weight and blood glucose in *Irs2*^*−/−*^;A7-Tg mice (Additional file 6: Figure S6). HFD-induced metabolic stress was tested by quantitative RT-PCR analysis of adipose tissue from the 15-month-old mice. Notably, HFD feeding increased mRNA levels of TNFα, Grp78/BiP and CHOP in *Irs2*^*−/−*^;A7-Tg mice (Fig. 6e).

Finally, we analyzed the effects of HFD on Aβ pathology of these mice. In the cerebral cortices of 10-month-old *Irs2*^*−/−*^;A7-Tg mice, HFD feeding increased the levels of soluble Aβ40 and Aβ42 (Fig. 6f). At this age, insoluble Aβ40 was significantly increased and insoluble Aβ42 also showed a trend of increase (*p* = 0.059) in HFD-fed *Irs2*^*−/−*^;A7-Tg cortices (Fig. 6f). Immunohistochemical analysis revealed that amyloid deposition, which was suppressed in *Irs2*^*−/−*^;A7-Tg compared with A7-Tg mice, was significantly enhanced in the cerebral neocortices of HFD-fed *Irs2*^*−/−*^;A7-Tg mice at 15 months of age (Fig. 6g). Altogether, HFD feeding of mice deficient in IRS-2 enhanced systemic inflammation and ER stress, exacerbated metabolic abnormalities, and promoted Aβ accumulation in the brain. These observations further support the direct role of diet-induced metabolic stress, the underpinnings of insulin resistance, in the induction and aggravation of AD-like Aβ pathology in the brain, irrespective of reduced insulin action per se.

## Discussion

The pathophysiological link between T2DM and AD is receiving increasing attention. Previous studies have suggested a possible involvement of insulin resistance, a core feature of T2DM, in the exacerbation of cognitive decline and pathology of AD [8, 16, 17]. In this study, we have addressed the questions on the chronological changes, causal relationships, and the molecular mechanisms of insulin resistance and amyloid pathology in a mouse model of AD with metabolic and genetic interventions.

In this study using A7-Tg mice, increased plasma insulin and blood glucose levels were observed as early as 2 months after initiation of HFD feeding on 3-month-old A7-Tg mice. In these mice, insulin-induced tyrosine phosphorylation of IR in the brain was suppressed compared to that in chow-fed mice (Fig. 1e). Defective brain insulin response was still evident in 9-month-old HFD-fed A7-Tg mice (Fig. 1f), indicating that chronic HFD exposure led to impaired insulin signaling both in the periphery and in the brain.

“Brain insulin resistance” could be due to either impaired response of brain cells to the extracellular insulin or disrupted transport of insulin to the brain [22]. Impaired response of brain cells was suggested by insulin stimulation assays of the brain tissues in vitro [8, 39]. With regard to the brain entry of insulin, the effect of metabolic overload on the insulin transport from blood to the brain parenchyma has remained unclear. We demonstrated that the ISF/plasma insulin ratio during the hyperinsulinemic clamp was reduced in HFD-fed A7-Tg mice (Fig. 1j). Decreased ratio of insulin in the cerebrospinal fluid (CSF) to plasma has previously been shown in insulin resistant conditions in humans and dogs [40–42], although it remains elusive if the changes in CSF recapitulate that in brain ISF. A few attempts to directly measure ISF insulin in the rat hypothalamus using the microdialysis technique showed that a meal or peripheral insulin infusion affected the levels of hypothalamic ISF insulin [43, 44]. It has also been shown in APP transgenic mice that an acute and modest elevation of the levels of peripheral insulin has no effect on the levels of ISF insulin [45]. However, it remained unknown whether the ISF insulin levels are altered under metabolic abnormalities within brain areas affected in AD, e.g., hippocampus and cerebral neocortices. Although multiple physiological processes other than insulin action might have been affected during the hyperinsulinemic clamp, and the quantitation of the basal ISF insulin levels should have enabled a more precise determination of the changes in brain insulin transport, our current observations under the condition of hyperinsulinemic clamp support the hypothesis that defective insulin transport to the brain might be one of the underlying mechanisms of diet-induced brain insulin resistance. Similar mechanisms have been suggested in the skeletal muscle, where HFD-induced impairment of insulin signaling in the endothelial cells contributed to the delayed insulin delivery to the interstitial space [46]. Whether impairment in insulin signaling in brain vessels contributes to the HFD-induced reduction in insulin transport to the brain should further be examined.

Time-course analyses in HFD-fed A7-Tg mice showed no changes in the levels of brain Aβ at 5 months of age when diet-induced insulin resistance was already observed, which was followed by increases in soluble and insoluble Aβ at 9 months of age (Fig. 2). These results suggested that the development of insulin resistance precedes that of Aβ accumulation. We therefore regarded the age of 9 months as the critical period of HFD-accelerated amyloid deposition in our A7-Tg model mice, when biochemical levels of brain Aβ, but not Aβ deposition, started to increase.

In view of the potential application of dietary interventions as therapeutic/preventive strategies for AD, it is critical to know whether T2DM-induced exacerbation of AD pathology is a reversible process. We therefore explored the efficacy of dietary interventions starting at a defined pathological stage of amyloid pathology in HFD-fed A7-Tg mice. Previously, caloric restriction and diet switching after HFD feeding have been shown to reduce amyloid deposition in AD model mice [36, 37, 47, 48]. In the current study, we systematically examined the effect of dietary interventions after the onset of HFD-induced insulin resistance and an increase in brain insoluble Aβ. Feeding the HFD followed by the chow diet significantly suppressed the effects of HFD feeding on insulin resistance and Aβ deposition, and HFD feeding followed by CR had more pronounced effects (Fig. 4). Altogether, these results suggested the positive and reversible correlation between the diet-induced peripheral/brain insulin resistance and brain amyloid pathology during the early stages of AD pathophysiology. The association between T2DM and amyloid pathology has been supported by studies focusing on the effects of insulin resistance in the late middle-aged population (45–64 years old) [18], with lesser effects in older population [49]. Thus, components of T2DM, e.g., insulin resistance, might affect the rate of Aβ deposition during the very early, preclinical stage of AD, which dovetails with our observation in our AD model mice that recapitulate the early stage of AD pathophysiology.

Previous studies in multiple AD mouse models have altogether shown the exacerbation of amyloid pathology by diet-induced metabolic abnormalities. In some models, changes in enzymes responsible for either Aβ degradation (IDE, neprilysin) or production (secretases) have been documented [19, 47, 50]. However, the mechanisms of increase in Aβ deposition were not consistently elucidated, and changes in the Aβ-related enzymes or APP fragments were not detected in our present study. We have for the first time examined the in vivo dynamics of brain ISF Aβ under HFD feeding using the brain microdialysis, and found that HFD feeding significantly extended the half-life of ISF Aβ in A7-Tg mice of 9-months-old, a critical age for Aβ accumulation (Fig. 3k). It has been suggested that the levels and the half-life of ISF Aβ might be affected by the presence of amyloid plaques in aged AD model mice [30, 51]; however, we might have minimized such an effect of insoluble Aβ pools that could affect the equilibrium with ISF Aβ in the plaque-free 9-month-old A7-Tg brains. No differences in ISF Aβ levels and clearance at 5 months of age was consistent with the lack of changes in Aβ levels by biochemical analysis (Fig. 3g), supporting the idea that decreased Aβ clearance that become evident by 9 months of age contributed to the accelerated amyloid pathology in the brains of our HFD-fed mice. In addition to the proteolytic digestion, the involvement of glial and vascular factors has been implicated in the clearance mechanisms of Aβ [52, 53]. Further study is needed to elucidate the molecular mechanism of diet-induced reduction in Aβ clearance.

Although a series of experiments in HFD-fed A7-Tg mice has suggested the causal relationship between insulin resistance and amyloid pathology, it is difficult to discriminate the effects of individual changes associated with diet-induced insulin resistance, e.g., hyperinsulinemia, hyperglycemia and reduced insulin action, on the Aβ pathology. To address this issue, we examined IRS-2-deficient A7-Tg mice as an alternative “insulin-resistant” mouse model. *Irs2*^*−/−*^ mice develop T2DM-like systemic phenotypes due to the reduced insulin action primarily in the liver and pancreatic β cells [28, 54]. IRS-2 expressed in the brain has been shown to play important roles in the regulation of food intake and peripheral insulin sensitivity, and could therefore be attributed to the development of diabetic phenotypes of *Irs2*^*−/−*^ mice [55]. Despite the pro-diabetic nature of the IRS-2 deletion, brains of *Irs2*^*−/−*^;A7-Tg mice accumulated lesser amount of amyloid plaques compared to that of A7-Tg mice (Fig. 5e), consistent with the earlier studies in Tg2576 mice lacking IRS-2 [23, 24], as well as in genetic disruption of neuronal insulin receptor [56] or IGF-1R [23, 26]. One possible mechanism underlying these alleviated phenotypes on amyloid deposition might be linked to the beneficial properties of reduced insulin/IGF-1 signaling for longevity, which have been extensively studied in a variety of organisms [57]. In contrast, insulin has been shown to increase the Aβ levels in cell culture studies [58, 59]. Overall, these lines of evidence suggest a suppressive role of reduced insulin signaling in Aβ accumulation. Another possible mechanism of the anti-amyloidgenic effect of *Irs2* deletion could be due to a compensatory activation of the other downstream pathway, i.e., IRS-1 signaling axis, although the functional diversity of signaling pathways via IRS-1 and IRS-2 in brains has not been fully characterized. Future experiments should reveal the specific changes in the downstream signaling of each IRS protein in the brains of *Irs2*^*−/−*^;A7-Tg mice.

Given the paradoxical results of the two independent “insulin resistant” AD mouse models, an important problem emerges whether the defective insulin signaling pathway is causative to the reduced amyloid pathology. To gain insights into the interplay between the genetically induced reduction in insulin signaling and metabolic stress, we have loaded HFD on *Irs2*^*−/−*^;A7-Tg mice and found an accelerating effect on amyloid accumulation in brains (Fig. 6f and g). The adverse effect of diet overloading on the development of insulin resistance was cumulative and not cancelled by IRS-2 deficiency. These findings led us to speculate that the diet-induced insulin resistance and its upstream causes, but not the reduced insulin action per se*,* is the mechanism accelerating the amyloid pathophysiology in vivo.

Among the known upstream events of impaired insulin signaling in metabolic overload or T2DM, we observed that the degrees of inflammation and ER stress signaling in adipose tissue were significantly elevated by HFD feeding (Fig. 5f). IRS-2-deficient A7-Tg mice developed diabetic phenotype without causing the elevation of inflammatory/stress responses, whereas HFD feeding increased the activity of these signaling pathways (Fig. 6e). Inflammation and ER stress are known to be key to the cause of insulin resistance [60], and recent studies have shown that the upregulation of inflammation and ER stress in the hypothalamus also contributes to the obesity-associated insulin resistance [61]. These inflammation and ER stress pathways have been implicated in the pathogenesis of AD as well [62]. Further study is needed to unravel the possible involvement of the diet-induced inflammatory and stress signaling in brains in the diet-induced exacerbation of amyloid pathophysiology.

## Conclusions

Our results suggest that diet-dependent, but not genetically-induced, insulin resistant states were causally and reversibly correlated with brain Aβ metabolism and amyloid formation during the early pathological stages of AD. These observations raise the possibility that the causal factors of insulin resistance, e.g., diet-induced metabolic stress, but not the impaired insulin signaling per se, might be directly involved in the induction of exacerbated amyloid pathology in the brain. Although strategies improving the insulin signal activity, e.g. intranasal insulin administration and insulin resistance ameliorating agents, are currently under clinical trials, our study underscores the early intervention into diet-induced metabolic abnormalities as promising preventive, disease-modifying therapeutic strategies against AD.

## Additional file

Additional file 1: **Figure S1.** Long-term exposure to HFD induces obesity, insulin resistance and hyperglycemia in A7-Tg mice. **a** Experimental scheme. A7-Tg mice were fed with a chow diet or HFD from 3 months of age. **b** Blood glucose levels of wild type (WT) and A7-Tg mice during the ITT (left) and the AUC of blood glucose (right) at 3 months of age (WT: *n* = 12; A7-Tg *n* = 11). **c** Monthly body weight changes of female A7-Tg mice (Chow: *n* = 10; HFD: *n* = 8). **d** Fasting blood glucose levels of 9-month-old female A7-Tg mice (Chow: *n* = 12; HFD: *n* = 15). **e** Blood glucose levels during the ITT and the AUC of blood glucose of 9-month-old female A7-Tg mice (Chow: *n* = 12; HFD: *n* = 14). **f** Fasting blood glucose levels of 18-month-old female A7-Tg mice (Chow: *n* = 11; HFD: *n* = 8). **g** Blood glucose levels during the ITT and the AUC of blood glucose of 18-month-old female A7-Tg mice (Chow: *n* = 11; HFD: *n* = 8). **h, i** 9-month-old male A7-Tg mice were intraperitoneally injected PBS or insulin and brain lysates were immunoprecipitaed with antibodies against IGF-1Rβ **(h)** and IRS-2 **(i)** followed by immunoblotting with anti-phospho Tyr (upper). Relative levels of signal intensity are shown (lower, Chow-PBS: *n* = 6; Chow-insulin: *n* = 6; HFD-PBS: *n* = 5; HFD-insulin: *n* = 6). Data are mean ± SEM. *p < 0.05, **p < 0.01, *** p < 0.001 (repeated-measures ANOVA with Sidak’s post-hoc test, **b, c, e, g**; unpaired *t* test, **b**, **d, e, f, g**; two-way ANOVA with Tukey’s post-hoc test, **h, i**). (DOCX 156 kb)

Additional file 2: **Figure S2.** HFD increases Aβ levels in the hippocampi of A7-Tg mice. Soluble Aβ levels in the hippocampus of 9-month-old male A7-Tg mice (Chow: *n* = 12; HFD: *n* = 11). Data are mean ± SEM. **p < 0.01 (unpaired *t* test). (DOCX 60 kb)

Additional file 3: **Figure S3.** HFD affects clearance of ISF Aβ. **a, b** The expression of APP, CTFα/β, BACE1 ADAM10 and α–tubulin was detected by immunoblots of cerebrocortical lysates of 5 (**a**, Chow: *n* = 9; HFD: *n* = 10) and 9 (**b**, Chow: *n* = 12; HFD: *n* = 11)-month-old A7-Tg mice (left). Relative levels of signal intensity are shown (right). **c** The expression of APP, sAPPtotal/α/β in lysates of 10-month-old acute brain slices of A7-Tg mice was detected by immunoblots and relative levels of signal intensity were measured (Chow: *n* = 8; HFD: *n* = 9). Data are mean ± SEM. **p < 0.01 (unpaired *t* test). (DOCX 105 kb)

Additional file 4: **Figure S4.** Caloric restriction improves insulin sensitivity and suppresses Aβ pathology in A7-Tg mice. **a** Monthly body weight changes of A7-Tg mice (Chow: *n* = 8; CR: *n* = 9). **b** Fasting blood glucose levels of 15-month-old A7-Tg mice (Chow: *n* = 12; CR: *n* = 11). **c** Blood glucose levels during the ITT (left) and the AUC of blood glucose (right) at 15 months of age (Chow: *n* = 12; CR: *n* = 11). **d** Levels of phosphorylated IR in A7-Tg mice fed with chow or CR upon insulin treatment. A7-Tg mice were intraperitoneally injected with PBS or insulin and Triton X-100-soluble cortical lysates were immunoprecipitaed with an anti-IR antibody followed by immunoblotting with anti-phospho Tyr and anti-IR antibodies at 15 month of age. Relative levels of signal intensity of phospho:total IR are shown (*n* = 6 per group). Data are mean ± SEM. *p < 0.05, ** *p* < 0.01, *** *p* < 0.001 (repeated-measures ANOVA with Sidak’s post-hoc test, **a, c**; two-way ANOVA with Tukey’s post-hoc test, **d**; unpaired *t* test, **b, c**). (DOCX 94 kb)

Additional file 5: **Figure S5.** Deletion of IRS-2 induces metabolic impairments without significant induction of inflammation and ER stress in adipose tissues of A7-Tg mice. Quantitative RT-PCR analysis of TNFα, Grp78/Bip and CHOP mRNA expression in the adipose tissues of 15-month-old *Irs2*^*+/+*^;A7-Tg mice (*n* = 5), HFD-fed *Irs2*^*+/+*^;A7-Tg mice (*n* = 6) or *Irs2*^*-/-*^;A7-Tg mice (*n* = 6). Data are mean ± SEM. *p < 0.05, ** *p* < 0.01, *** *p* < 0.001 (one-way ANOVA with Tukey’s post-hoc test). (DOCX 70 kb)

Additional file 6: **Figure S6.** HFD feeding on IRS-2-deficient A7-Tg mice exacerbates diabetic phenotype. **a** Effect of HFD feeding on body weight of female *Irs2*^*-/-*^;A7-Tg mice (*n* = 6 per group). **b** Effect of HFD feeding on blood glucose levels of female *Irs2*^*-/-*^;A7-Tg mice (*n* = 6 per group). Data are mean ± SEM. *p < 0.05, **p < 0.01, *** *p* < 0.001 (repeated-measures ANOVA with Sidak’s post-hoc test). (DOCX 73 kb)

## Abbreviations

AD: Alzheimer’s disease
Aβ: Amyloid-β peptide
CR: Caloric restriction
CSF: Cerebrospinal fluid
ER: Endoplasmic reticulum
HFD: High-fat diet
IDE: Insulin degrading enzyme
IR: Insulin receptor
IRS: Insulin receptor substrate
ISF: Interstitial fluid
MWCO: Molecular weight cut-off
T2DM: Type 2 diabetes mellitus
