# Supplementary material for: Differential effects of diet- and genetically-induced brain insulin resistance on amyloid pathology in a mouse model of Alzheimer’s disease
Source: Mol Neurodegener. 2019 Apr 12;14:15. doi: 10.1186/s13024-019-0315-7 (PMC6460655; doi:10.1186/s13024-019-0315-7)
Supplement: Supplementary file 1 — Figure S1. Long-term exposure to HFD induces obesity, insulin resistance and hyperglycemia in A7-Tg mice. a Experimental scheme. A7-Tg mice were fed with a chow diet or HFD from 3 months of age. b Blood glucose levels of wild type (WT) and A7-Tg mice during the ITT (left) and the AUC of blood glucose (right) at 3 months of age (WT: n = 12; A7-Tg n = 11). c Monthly body weight changes of female A7-Tg mice (Chow: n = 10; HFD: n = 8). d Fasting blood glucose levels of 9-month-old female A7-Tg mice (Chow: n = 12; HFD: n = 15). e Blood glucose levels during the ITT and the AUC of blood glucose of 9-month-old female A7-Tg mice (Chow: n = 12; HFD: n = 14). f Fasting blood glucose levels of 18-month-old female A7-Tg mice (Chow: n = 11; HFD: n = 8). g Blood glucose levels during the ITT and the AUC of blood glucose of 18-month-old female A7-Tg mice (Chow: n = 11; HFD: n = 8). h, i 9-month-old male A7-Tg mice were intraperitoneally injected PBS or insulin and brain lysates were immunoprecipitaed with antibodies against IGF-1Rβ (h) and IRS-2 (i) followed by immunoblotting with anti-phospho Tyr (upper). Relative levels of signal intensity are shown (lower, Chow-PBS: n = 6; Chow-insulin: n = 6; HFD-PBS: n = 5; HFD-insulin: n = 6). Data are mean ± SEM. *p < 0.05, **p < 0.01, *** p < 0.001 (repeated-measures ANOVA with Sidak’s post-hoc test, b, c, e, g; unpaired t test, b, d, e, f, g; two-way ANOVA with Tukey’s post-hoc test, h, i). (DOCX 156 kb) [file 13024_2019_315_MOESM1_ESM.docx]

**Additional file 1: Figure S1. Long-term exposure to HFD induces obesity, insulin resistance and hyperglycemia in A7-Tg mice. a** Experimental scheme. A7-Tg mice were fed with a chow diet or HFD from 3 months of age. **b** Blood glucose levels of wild type (WT) and A7-Tg mice during the ITT (left) and the AUC of blood glucose (right) at 3 months of age (WT: *n* = 12; A7-Tg *n* = 11). **c** Monthly body weight changes of female A7-Tg mice (Chow: *n* = 10; HFD: *n* = 8). **d** Fasting blood glucose levels of 9-month-old female A7-Tg mice (Chow: *n* = 12; HFD: *n* = 15). **e** Blood glucose levels during the ITT and the area AUC of blood glucose of 9-month-old female A7-Tg mice (Chow: *n* = 12; HFD: *n* = 14). **f** Fasting blood glucose levels of 18-month-old female A7-Tg mice (Chow: *n* = 11; HFD: *n* = 8). **g** Blood glucose levels during the ITT and the area AUC of blood glucose of 18-month-old female A7-Tg mice (Chow: *n* = 11; HFD: *n* = 8). **h, i** 9-month-old male A7-Tg mice were intraperitoneally injected PBS or insulin and brain lysates were immunoprecipitaed with antibodies against IGF-1Rβ **(h)** and IRS-2 **(i)** followed by immunoblotting with anti-phospho Tyr (upper). Relative levels of signal intensity are shown (lower, Chow-PBS: *n* = 6; Chow-insulin: *n* = 6; HFD-PBS: *n* = 5; HFD-insulin: *n* = 6). Data are mean ± SEM. *p < 0.05, **p < 0.01, *** p < 0.001 (repeated-measures ANOVA with Sidak’s post-hoc test, **b, c, e, g**; unpaired *t* test, **b**, **d, e, f, g**; two-way ANOVA with Tukey’s post-hoc test, **h, i**).
