# Supplementary material for: Differential effects of diet- and genetically-induced brain insulin resistance on amyloid pathology in a mouse model of Alzheimer’s disease
Source: Mol Neurodegener. 2019 Apr 12;14:15. doi: 10.1186/s13024-019-0315-7 (PMC6460655; doi:10.1186/s13024-019-0315-7)
Supplement: Supplementary file 2 — Figure S2. HFD increases Aβ levels in the hippocampi of A7-Tg mice. Soluble Aβ levels in the hippocampus of 9-month-old male A7-Tg mice (Chow: n = 12; HFD: n = 11). Data are mean ± SEM. **p < 0.01 (unpaired t test). (DOCX 60 kb) [file 13024_2019_315_MOESM2_ESM.docx]

**Additional file 2: Figure S2. HFD increases Aβ levels in the hippocampi of A7-Tg mice.**

Soluble Aβ levels in the hippocampus of 9-month-old male A7-Tg mice (Chow: *n* = 12; HFD: *n* = 11). Data are mean $\pm$ SEM. ***p* < 0.01 (unpaired *t* test).
