# Supplementary material for: Differential effects of diet- and genetically-induced brain insulin resistance on amyloid pathology in a mouse model of Alzheimer’s disease
Source: Mol Neurodegener. 2019 Apr 12;14:15. doi: 10.1186/s13024-019-0315-7 (PMC6460655; doi:10.1186/s13024-019-0315-7)
Supplement: Supplementary file 3 — Figure S3. HFD affects clearance of ISF Aβ. a, b The expression of APP, CTFα/β, BACE1 ADAM10 and α–tubulin was detected by immunoblots of cerebrocortical lysates of 5 (a, Chow: n = 9; HFD: n = 10) and 9 (b, Chow: n = 12; HFD: n = 11)-month-old A7-Tg mice (left). Relative levels of signal intensity are shown (right). c The expression of APP, sAPPtotal/α/β in lysates of 10-month-old acute brain slices of A7-Tg mice was detected by immunoblots and relative levels of signal intensity were measured (Chow: n = 8; HFD: n = 9). Data are mean ± SEM. **p < 0.01 (unpaired t test). (DOCX 105 kb) [file 13024_2019_315_MOESM3_ESM.docx]

**Additional file 3: Figure S3. HFD affects clearance of ISF Aβ. a, b** The expression of APP, CTFα/β, BACE1 ADAM10 and α–tubulin was detected by immunoblots of cerebrocortical lysates of 5 (**a**, Chow: *n* = 9; HFD: *n* = 10) and 9 (**b**, Chow: *n* = 12; HFD: *n* = 11) month-old A7-Tg mice (left). Relative levels of signal intensity are shown (right). **c** The expression of APP, sAPPtotal/α/β in lysates of 10-month-old acute brain slices of A7-Tg mice was detected by immunoblots and relative levels of signal intensity was measured (Chow: *n* = 8; HFD: *n* = 9). Data are mean $\pm$ SEM. ***p* < 0.01 (unpaired *t* test).
