# Supplementary material for: Differential effects of diet- and genetically-induced brain insulin resistance on amyloid pathology in a mouse model of Alzheimer’s disease
Source: Mol Neurodegener. 2019 Apr 12;14:15. doi: 10.1186/s13024-019-0315-7 (PMC6460655; doi:10.1186/s13024-019-0315-7)
Supplement: Supplementary file 4 — Figure S4. Caloric restriction improves insulin sensitivity and suppresses Aβ pathology in A7-Tg mice. a Monthly body weight changes of A7-Tg mice (Chow: n = 8; CR: n = 9). b Fasting blood glucose levels of 15-month-old A7-Tg mice (Chow: n = 12; CR: n = 11). c Blood glucose levels during the ITT (left) and the AUC of blood glucose (right) at 15 months of age (Chow: n = 12; CR: n = 11). d Levels of phosphorylated IR in A7-Tg mice fed with chow or CR upon insulin treatment. A7-Tg mice were intraperitoneally injected with PBS or insulin and Triton X-100-soluble cortical lysates were immunoprecipitaed with an anti-IR antibody followed by immunoblotting with anti-phospho Tyr and anti-IR antibodies at 15 month of age. Relative levels of signal intensity of phospho:total IR are shown (n = 6 per group). Data are mean ± SEM. *p < 0.05, ** p < 0.01, *** p < 0.001 (repeated-measures ANOVA with Sidak’s post-hoc test, a, c; two-way ANOVA with Tukey’s post-hoc test, d; unpaired t test, b, c). (DOCX 94 kb) [file 13024_2019_315_MOESM4_ESM.docx]

**Additional file 4: Figure S4. Dietary interventions reverse HFD-induced metabolic impairments and accelerated Aβ pathology in A7-Tg mice. a** Monthly body weight changes of A7-Tg mice (Chow: *n* = 8; CR: *n* = 9). **b** Fasting blood glucose levels of 15-month-old A7-Tg mice (Chow: *n* = 12; CR: *n* = 11). **c** Blood glucose levels during the ITT (left) and the AUC of blood glucose (right) at 15 months of age (Chow: *n* = 12; CR: *n* = 11). **d** Levels of phosphorylated IR in A7-Tg mice fed with chow or CR upon insulin treatment. A7-Tg mice were intraperitoneally injected with PBS or insulin and Triton X-100-soluble cortical lysates were immunoprecipitaed with an anti-IR antibody followed by immunoblotting with anti-phospho Tyr and anti-IR antibodies at 15 month of age. Relative levels of signal intensity of phospho:total IR are shown (*n* = 6 per group). Data are mean $\pm$ SEM. **p* < 0.05, ** *p* < 0.01, *** *p* < 0.001 (repeated-measures ANOVA with Sidak’s post-hoc test, **a, c**; two-way ANOVA with Tukey’s post-hoc test, **d**; unpaired *t* test, **b, c**).
