# Supplementary material for: Differential effects of diet- and genetically-induced brain insulin resistance on amyloid pathology in a mouse model of Alzheimer’s disease
Source: Mol Neurodegener. 2019 Apr 12;14:15. doi: 10.1186/s13024-019-0315-7 (PMC6460655; doi:10.1186/s13024-019-0315-7)
Supplement: Supplementary file 5 — Figure S5. Deletion of IRS-2 induces metabolic impairments without significant induction of inflammation and ER stress in adipose tissues of A7-Tg mice. Quantitative RT-PCR analysis of TNFα, Grp78/Bip and CHOP mRNA expression in the adipose tissues of 15-month-old Irs2+/+;A7-Tg mice (n = 5), HFD-fed Irs2+/+;A7-Tg mice (n = 6) or Irs2-/-;A7-Tg mice (n = 6). Data are mean ± SEM. *p < 0.05, ** p < 0.01, *** p < 0.001 (one-way ANOVA with Tukey’s post-hoc test). (DOCX 70 kb) [file 13024_2019_315_MOESM5_ESM.docx]

Additional file 5: **Figure S5.** Deletion of IRS-2 induces metabolic impairments without significant induction of inflammation and ER stress in adipose tissues of A7-Tg mice. Quantitative RT-PCR analysis of TNFα, Grp78/Bip and CHOP mRNA expression in the adipose tissues of 15-month-old *Irs2^+/+^*;A7-Tg mice (*n* = 5), HFD-fed *Irs2^+/+^*;A7-Tg mice (*n* = 6) or *Irs2^-/-^*;A7-Tg mice (*n* = 6). Data are mean $\pm$ SEM. **p* < 0.05, ** *p* < 0.01, *** *p* < 0.001 (one-way ANOVA with Tukey’s post-hoc test).
