# Supplementary material for: Differential effects of diet- and genetically-induced brain insulin resistance on amyloid pathology in a mouse model of Alzheimer’s disease
Source: Mol Neurodegener. 2019 Apr 12;14:15. doi: 10.1186/s13024-019-0315-7 (PMC6460655; doi:10.1186/s13024-019-0315-7)
Supplement: Supplementary file 6 — Figure S6. HFD feeding on IRS-2-deficient A7-Tg mice exacerbates diabetic phenotype. a Effect of HFD feeding on body weight of female Irs2-/-;A7-Tg mice (n = 6 per group). b Effect of HFD feeding on blood glucose levels of female Irs2-/-;A7-Tg mice (n = 6 per group). Data are mean ± SEM. *p < 0.05, **p < 0.01, *** p < 0.001 (repeated-measures ANOVA with Sidak’s post-hoc test). (DOCX 73 kb) [file 13024_2019_315_MOESM6_ESM.docx]

Additional file 6: **Figure S6.** HFD feeding on IRS-2-deficient A7-Tg mice exacerbates diabetic phenotype. **a** Effect of HFD feeding on body weight of female *Irs2^-/-^*;A7-Tg or *Irs2^-/-^*;A7-Tg mice (*n* = 6 per group). **b** Effect of HFD feeding on blood glucose levels of female *Irs2^-/-^*;A7-Tg or *Irs2^-/-^*;A7-Tg mice (*n* = 6 per group). Data are mean $\pm$ SEM. **p* < 0.05, ***p* < 0.01, *** *p* < 0.001 (repeated-measures ANOVA with Sidak’s post-hoc test).
